# Standard Versus Family-Based Screening, Brief Intervention, and Referral to Treatment for Adolescent Substance Use in Primary Care: Protocol for a Multisite Randomized Effectiveness Trial

**DOI:** 10.2196/54486

**Published:** 2024-05-31

**Authors:** Aaron Hogue, Nicole P Porter, Timothy J Ozechowski, Sara J Becker, Megan A O'Grady, Molly Bobek, Monica Cerniglia, Kevin Ambrose, Alexandra MacLean, Scott E Hadland, Hetty Cunningham, Sarah M Bagley, Lon Sherritt, Maddie O'Connell, Lydia A Shrier, Sion Kim Harris

**Affiliations:** 1 Family and Adolescent Clinical Technology & Science Partnership to End Addiction New York, NY United States; 2 Abt Associates Cambridge, MA United States; 3 Center for Dissemination and Implementation Science Northwestern University Feinberg School of Medicine Chicago, IL United States; 4 University of Connecticut Health Center Farmington, CT United States; 5 Division of Adolescent and Young Adult Medicine Massachusetts General Hospital for Children Boston, MA United States; 6 Department of Pediatrics Harvard Medical School Boston, MA United States; 7 Department of Pediatrics, Vagelos College of Physicians and Surgeons Columbia University Irving Medical Center NewYork-Presbyterian Hospital New York, NY United States; 8 Department of Pediatrics Chobanian & Avedisian School of Medicine Boston, MA United States; 9 Grayken Center for Addiction Boston Medical Center Boston, MA United States; 10 Cornerstone Systems Northwest Lynden, WA United States; 11 Division of Adolescent/Young Adult Medicine Boston Children's Hospital Boston, MA United States

**Keywords:** adolescent substance use, pediatric primary care, screening, brief intervention, referral to treatment, family-based

## Abstract

**Background:**

Screening, brief intervention, and referral to treatment for adolescents (SBIRT-A) is widely recommended to promote detection and early intervention for alcohol and other drug (AOD) use in pediatric primary care. Existing SBIRT-A procedures rely almost exclusively on adolescents alone, despite the recognition of caregivers as critical protective factors in adolescent development and AOD use. Moreover, controlled SBIRT-A studies conducted in primary care have yielded inconsistent findings about implementation feasibility and effects on AOD outcomes and overall developmental functioning. There is urgent need to investigate the value of systematically incorporating caregivers in SBIRT-A procedures.

**Objective:**

This randomized effectiveness trial will advance research and scope on SBIRT-A in primary care by conducting a head-to-head test of 2 conceptually grounded, evidence-informed approaches: a standard adolescent-only approach (SBIRT-A-Standard) versus a more expansive family-based approach (SBIRT-A-Family). The SBIRT-A-Family approach enhances the procedures of the SBIRT-A-Standard approach by screening for AOD risk with both adolescents and caregivers; leveraging multidomain, multireporter AOD risk and protection data to inform case identification and risk categorization; and directly involving caregivers in brief intervention and referral to treatment activities.

**Methods:**

The study will include 2300 adolescents (aged 12-17 y) and their caregivers attending 1 of 3 hospital-affiliated pediatric settings serving diverse patient populations in major urban areas. Study recruitment, screening, randomization, and all SBIRT-A activities will occur during a single pediatric visit. SBIRT-A procedures will be delivered digitally on handheld tablets using patient-facing and provider-facing programming. Primary outcomes (AOD use, co-occurring behavior problems, and parent-adolescent communication about AOD use) and secondary outcomes (adolescent quality of life, adolescent risk factors, and therapy attendance) will be assessed at screening and initial assessment and 3-, 6-, 9-, and 12-month follow-ups. The study is well powered to conduct all planned main and moderator (age, sex, race, ethnicity, and youth AOD risk status) analyses.

**Results:**

This study will be conducted over a 5-year period. Provider training was initiated in year 1 (December 2023). Participant recruitment and follow-up data collection began in year 2 (March 2024). We expect the results from this study to be published in early 2027.

**Conclusions:**

SBIRT-A is widely endorsed but currently underused in pediatric primary care settings, and questions remain about optimal approaches and overall effectiveness. In particular, referral to treatment procedures in primary care remains virtually untested among youth. In addition, whereas research strongly supports involving families in interventions for adolescent AOD, SBIRT-A effectiveness trial testing approaches that actively engage family members in primary care are absent. This trial is designed to help fill these research gaps to inform the critical health decision of whether and how to include caregivers in SBIRT-A activities conducted in pediatric primary care.

**Trial Registration:**

ClinicalTrials.gov NCT05964010; https://www.clinicaltrials.gov/study/NCT05964010

**International Registered Report Identifier (IRRID):**

PRR1-10.2196/54486

## Introduction

### Alcohol and Other Drug Use Problems Exert Enormous Negative Impacts on Adolescents

The level of unmet intervention need among adolescents with risky alcohol and other drug (AOD) use is a persistent public health problem. Recent data from the 2023 Monitoring the Future national survey indicate that, among 12th graders, 46% reported using alcohol (more than just a few sips); 39% marijuana; 39% nicotine via vaping; and 43% illicit drugs, including inhalants [1]. According to the 2023 National Survey on Drug Use and Health (NSDUH), among adolescents aged 12 to 17 years in 2022, 8.7% (or 2,228,000 adolescents) reported a substance use disorder in the past year [2]. Troublingly, the rates of treatment enrollment among this high-need age group are considerably low, with only 4.6% of the adolescents who meet the criteria for substance use disorders receiving specialty care [2]. When left unaddressed or ineffectively treated, AOD use during adolescence often continues into adulthood, precipitating long-term substance use and mental health disorders and being associated with a cascade of other health consequences, as well as imparting enormous economic costs to society [3].

Preventing youth AOD use and intervening early with those who use are key to reducing the many consequences of risky use. As shown in neurodevelopment research on risky behavior in youth, adolescence is the critical period both for starting AOD use and experiencing harmful consequences as a result [4]. Adolescence is a developmental stage characterized by risk behaviors, including AOD experimentation; adolescents are also vulnerable to negative impacts of addictive substances, which can further impair judgment, interfere with brain development, and increase addiction risk [5]. Moreover, AOD use is associated with the 3 leading causes of death among teenagers: motor vehicle crashes, homicides, and suicides [6]. Prevention and early intervention can also prevent progression to more severe AOD use that carries the risk of overdose and death.

### Screening, Brief Intervention, and Referral to Treatment for Adolescents Is Widely Recommended for Addressing AOD Use in Primary Care

Given that the majority of teenagers in the United States visit primary care (PC) clinics at least once per year [7], PC settings provide rich opportunities to detect and intervene with adolescents at risk for AOD problems. The most recent guidelines issued by the American Academy of Pediatrics prescribe universal AOD screening for teenagers during both routine preventive appointments and nonpreventive PC visits [8]. Recognizing the unique role that PC providers can play in meeting the challenge of adolescent AOD use, the American Academy of Pediatrics further recommends that providers learn procedures for screening, brief intervention, and referral to treatment (SBIRT) and how to integrate SBIRT into medical care for adolescents [9]. SBIRT for adolescents (SBIRT-A) entails a set of clinical procedures for detecting youth at risk of AOD problems and delivering appropriate prevention or early intervention, including universal screening for AOD risk level and formulaic guidelines for providing brief motivational interventions and appropriate treatment referral to patients identified as at risk [8,10]. Notably, when delivered using standard procedures, SBIRT-A amounts to an adolescent-focused approach in which most or all activities involve only the given youth [11,12].

SBIRT-A is widely recommended to promote detection and early intervention for AOD use in PC [9,13]. In practice, although 50% to 86% of pediatricians report screening for AOD use, the use of standardized screening assessments and systematic brief interventions (BIs) is low [14]. In research trials, approximately 25% to 35% of adolescents screen positive for substance use when systematic screening is implemented [15,16]. Research reviews and meta-analyses show that BI procedures can be effective in reducing adolescent drinking [17,18] and, when delivered in PC settings, have demonstrable impacts and cost-effectiveness for quality-of-life indicators [18-20]. Recent studies, including 2 multisite effectiveness trials that collectively examined thousands of adolescent visits in PC clinics [15,21], further suggest that SBIRT-A is scalable in PC [16,22].

Despite these promising developments, there remain significant limitations in SBIRT-A research and practice in PC. Prior controlled SBIRT-A studies conducted in PC have yielded inconsistent findings with regard to implementation feasibility [23], in large part due to significant practice barriers to delivering SBIRT-A in pediatric care. PC providers are generally reluctant to implement SBIRT-A, citing insufficient time, limited training in substance use, unfamiliarity with the procedures, a lack of readiness to address a positive AOD screen result, limited resources for referrals to specialty care, and concerns about confidentiality [24-28]. Moreover, despite data from systematic reviews supporting BIs in PC [18], the US Preventive Services Task Force recently determined that evidence is insufficient to assess the balance of benefits versus harms of screening adolescents for unhealthy AOD use due to variability in study methods and quality [29]; consequently, it withheld a recommendation for widespread SBIRT-A delivery in pediatric settings [10,30]. Given existing gaps in the SBIRT-A research base on the effectiveness of SBI and the effect of involving caregivers in SBI procedures as well as the impact of BIs on outcomes beyond AOD use and the corresponding uncertainties in the policy and practice status of SBIRT-A in pediatric settings, there is urgent need to advance the scope of SBIRT-A effectiveness studies in PC [28].

### Tablet-Guided SBIRT-A Procedures Have the Potential to Reduce Implementation Barriers

Tablet-guided SBIRT-A approaches have the potential to reduce documented implementation barriers through offering a standardized road map for addressing AOD use. Tablet-guided SBIRT-A procedures eliminate the need for the memorization of complicated adjunctive SBIRT-A procedures. Instead, tablet-guided SBIRT-A approaches provide a new and systematic way to address positive AOD screening that is more efficient than the existing eclectic practices that providers currently rely on. Providers are guided through step-by-step interventions designed with their time constraints in mind, leveraging automated tools and well-vetted workflow integration [16,31,32]. Procedures include recommendations for providers on addressing positive AOD screens with youth and their caregivers without jeopardizing youth autonomy or violating confidentiality.

### Incorporating Caregivers in SBIRT-A Procedures Has the Potential to Enhance Effectiveness

#### Overview

The standard SBIRT-A approach focuses almost exclusively on the adolescent patient by including only the given youth in screening and intervention procedures [11]. Nevertheless, the SBIRT-A evidence base indicates a need for improvements in effectiveness and feasibility in PC [33,34]. Incorporating caregivers in SBIRT-A activities could enhance the effectiveness of the approach in several ways, as outlined in the following subsections.

#### Broaden the Screening Search

The “no missed opportunities” paradigm [35] recognizes caregivers as vital sources of information about adolescent AOD risk. Using multiple sources of information to assess youth AOD use is more accurate than relying on any single source [36]. Studies suggest that caregiver reports are fair to good proxy measures of youth AOD behavior [37], although they typically underestimate consumption [38]. Caregiver report may be particularly useful when youth have minimized self-report of AOD use or impairment. Involving caregivers in screening has the potential to boost the accuracy of problem identification and also set the stage for their involvement in BI and referral to treatment (RT) procedures [39].

#### Widen the Screening Net

In addition to assessing AOD use, screening procedures can collect supplemental data to generate a multidomain profile of AOD risk. The screening net can be widened in at least 2 ways. First, caregivers can complete screening tools for youth co-occurring behavior problems that both signal and exacerbate AOD risk: anxiety and depression, impulsivity and sensation seeking, and aggression and conduct problems [4,40]. Second, caregivers as well as youth can provide valuable data on family strengths, including key protective factors such as parent-adolescent communication about AOD use [41,42]. Data on these supplemental risk and protective factors can be converted into “risk algorithms” that more accurately codify AOD risk profiles, boosting both the acuity of risk screening and the attunement of subsequent SBIRT-A activities [43].

#### Deepen BI Impact

Developmental science asserts that supportive familial networks are potent protections against individual-level developmental processes that predispose adolescents to AOD and other risky behavior. BIs for adolescent AOD use that incorporate caregivers have shown their mettle [44] and are likely to have considerable added value over adolescent-only BIs in PC settings [45], especially given that caregivers who harbor AOD and related behavioral concerns for their children often seek anticipatory guidance from PC providers [27].

#### Strengthen RT Linkages

Influencing teenagers seeking medical care to enroll in AOD services typically requires caregiver involvement [39]. Nevertheless, there are numerous challenges to engaging caregivers in treatment referral, including caregiver skepticism about the value of AOD services, the stigma and fear of being judged for the youth’s problems, and hopelessness about the possibility of change [46]. PC providers are well positioned to shape caregiver attitudes and behaviors concerning treatment referral, given their authority on health care issues. Providers can explain AOD risks and ramifications, acknowledge past efforts to address AOD and related behaviors, and reinforce the importance of caregiver influence on adolescent well-being along with the possibility of behavior change—techniques common to family-focused treatment engagement models [47]. Providers can also join caregivers in talking directly with their teenagers about AOD risk, using evidence-based strategies [48] to mobilize caregiver capacity to facilitate youth enrolling in AOD services.

### Specific Aims of the Randomized Effectiveness Trial

This randomized effectiveness trial will advance research on SBIRT-A in PC by conducting a head-to-head test of 2 conceptually grounded, evidence-informed approaches: a standard adolescent-only approach (SBIRT-A-Standard) versus a more expansive family-based approach (SBIRT-A-Family). The SBIRT-A-Family approach enhances the procedures of the SBIRT-A-Standard approach by screening for AOD risk with both adolescents and caregivers; leveraging multidomain, multireporter AOD risk and protection data to inform case identification and risk categorization; and directly involving caregivers in BI and RT activities [43]. The trial will test the effectiveness of SBIRT-A-Standard versus SBIRT-A-Family for preventing the escalation of AOD use (aim 1); reducing AOD use risk factors and consequences, decreasing co-occurring behavior problems, enhancing parent-adolescent communication about AOD use, and increasing quality-of-life indicators (aim 2); linking youth and families to behavior counseling services (aim 3); and investigating the extent to which the effects of SBIRT-A-Family vary based on youth characteristics (age, sex, and race or ethnicity) and youth AOD risk status (aim 4).

## Methods

### Trial Design

This trial uses a 2-arm parallel-group multisite randomized effectiveness design. The 2 SBIRT-A approaches (SBIRT-A-Standard and SBIRT-A-Family) both use digitally supported BIs [16,49] that are a substantial upgrade over routine practice and consistent with the recommendations of the American Academy of Pediatrics to leverage digital tools. Individual youth and their caregivers are randomized as a family unit to the study condition. Adolescent and family outcomes for 2300 youth (n=2000, 86.96% identified as at risk of AOD use and n=300, 13.04% as a developmental comparison identified as low risk) are assessed over 1-year follow-up.

The protocol was developed according to the SPIRIT (Standard Protocol Items: Recommendations for Interventional Trials) 2013 statement (Multimedia Appendix 1).

### Study Participants

#### Study Sites and Providers

The study occurs in 3 hospital-affiliated pediatric settings: Mass General for Children, Boston Medical Center, and Columbia University Irving Medical Center. All 3 settings are in major urban areas that serve a diverse range of patients. In aggregate, these 3 settings receive approximately 20,000 visits annually from youth in the targeted age range (12-17 y); the sites reported that 10% to 15% of age-targeted youth routinely identify as positive for AOD use via existing site screening methods. All sites currently screen youth routinely for AOD risk via digital screening methods and expressed intention to adopt BIs. All site staff (eg, physicians, nurse practitioners, and behavioral specialists) who provide BI and RT services to adolescents are eligible to provide SBIRT-A services as part of this trial. Physicians in leadership roles at each site (eg, the medical director) will serve as site champions to pioneer the implementation of site-based study procedures and promote acceptance and buy-in among all site staff [50]. Each site will receive performance feedback (eg, the uptake of SBIRT procedures and progress toward patient enrollment goals) and participate in cross-site monthly learning collaboratives to discuss and problem-solve implementation barriers and facilitators. The study team will partner with participating providers to revise implementation procedures as needed to enhance the uptake of SBIRT procedures.

#### Study Patients and Inclusion Criteria

The study enrolls adolescents and their caregivers attending a PC visit at 1 of the 3 study sites. The study inclusion criteria are as follows: youth aged 12 to 17 years; a primary caregiver (ie, parental figure) also in attendance; youth is fluent in English, and caregiver is fluent in English or Spanish; both are capable of using audio-assisted informed consent procedures and independently operating a handheld tablet; and both complete routine site AOD risk screening questions prompted during PC visit intake. On the basis of existing patient flow at each site, we have projected aggregate self-reported demographic characteristics of enrolled youth (Table 1).

This profile supports planned moderator analyses for sex, race, ethnicity, and age subgroups. The randomization procedures will (1) block and stratify by clinic site and (2) use imbalance minimization procedures for adolescent age (12-14 y vs 15-17 y) and study clinician.

**Table 1:** Projected aggregate self-reported demographic characteristics of enrolled youth (N=2000).

Demographic characteristics			Participants, n (%)
**Sex**			
	Male	1000 (50)	
	Female	1000 (50)	
**Race and ethnicity**			
	Black	620 (31)	
	Latinx	580 (29)	
	White	680 (34)	
	Other	120 (6)	
**Age**			
	12 to 14 years	1100 (55)	
	15 to 17 years	900 (45)	

### Recruitment, Randomization, Intervention Flow, and Outcome Assessment Procedures

**Figure 1:**
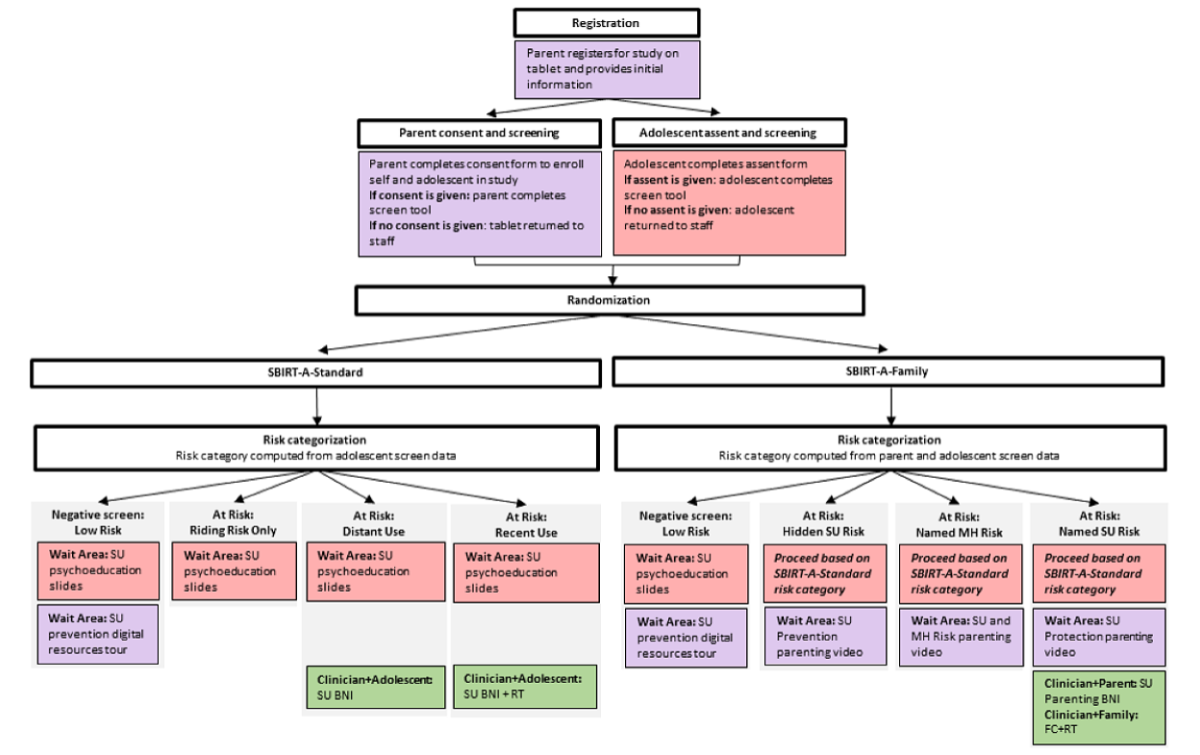
Patient flow diagram of the screening, brief intervention, and referral to treatment for adolescents: standard adolescent-only approach (SBIRT-A-Standard) versus SBIRT-A: family-based approach (SBIRT-A-Family) for adolescent substance use (SU) in the primary care multisite randomized effectiveness trial: recruitment, screening, randomization, and intervention recommendations. BNI: brief negotiated interview; FC: facilitated conversation; MH: mental health; RT: referral to treatment.

Study recruitment and randomization as well as all SBIRT-A activities occur on the day of the visit in PC offices. Figure 1 depicts the flow of patient recruitment, patient randomization, and SBIRT-A delivery for various risk categories. Recruitment efforts will be multimethod, consisting of (1) informational flyers in wait areas, (2) outreach to families by site research staff, and (3) warm handoff by site administrators and providers when needed. Interested caregivers are prompted to contact on-site research staff, who then give the designated caregiver a tablet to (1) complete an electronic research consent form, (2) provide family demographic and brief assessment information, and (3) complete a screening tool assessing their concerns about the adolescent’s AOD use and related behavior problems as well as family communication about AOD issues. Simultaneously, research staff give a tablet to the youth, who completes an electronic research assent form, and if their caregiver gives consent, an AOD screening tool and brief assessment information.

For families in which both caregiver and youth give informed consent and assent and complete screening, the tablet automatically (1) randomizes the family to study condition, using urn randomization to achieve balanced allocation of participants based on sex, race, ethnicity, and age group; and (2) computes a risk category. In SBIRT-A-Standard, the risk category (*low risk*, *riding risk only*, *distant use*, or *recent use*) is assigned using youth screening data only, while SBIRT-A-Family uses youth plus caregiver screening data to assign the risk category (*low risk*, *hidden substance use risk*, *named mental health risk*, or *named substance use risk*). For youth and families assigned to the *low risk* category (ie, no positive screen result), youth are invited to view tablet-delivered psychoeducation slides, and caregivers are invited to view a video tour and enrollment link to web-based educational and support resources for adolescent AOD. For youth and families assigned to an *at risk* category, youth and caregivers each view tablet-delivered psychoeducation videos and (for some risk categories) complete provider-facing BI and RT activities that are differentiated based on condition assignment and tailored to the respective risk categories. Multimedia Appendix 2 summarizes study activities for each condition; full descriptions are provided in the Study Conditions subsection. All study providers are trained to deliver both SBIRT-A approaches (ie, provider crossover design).

**Figure 2:**
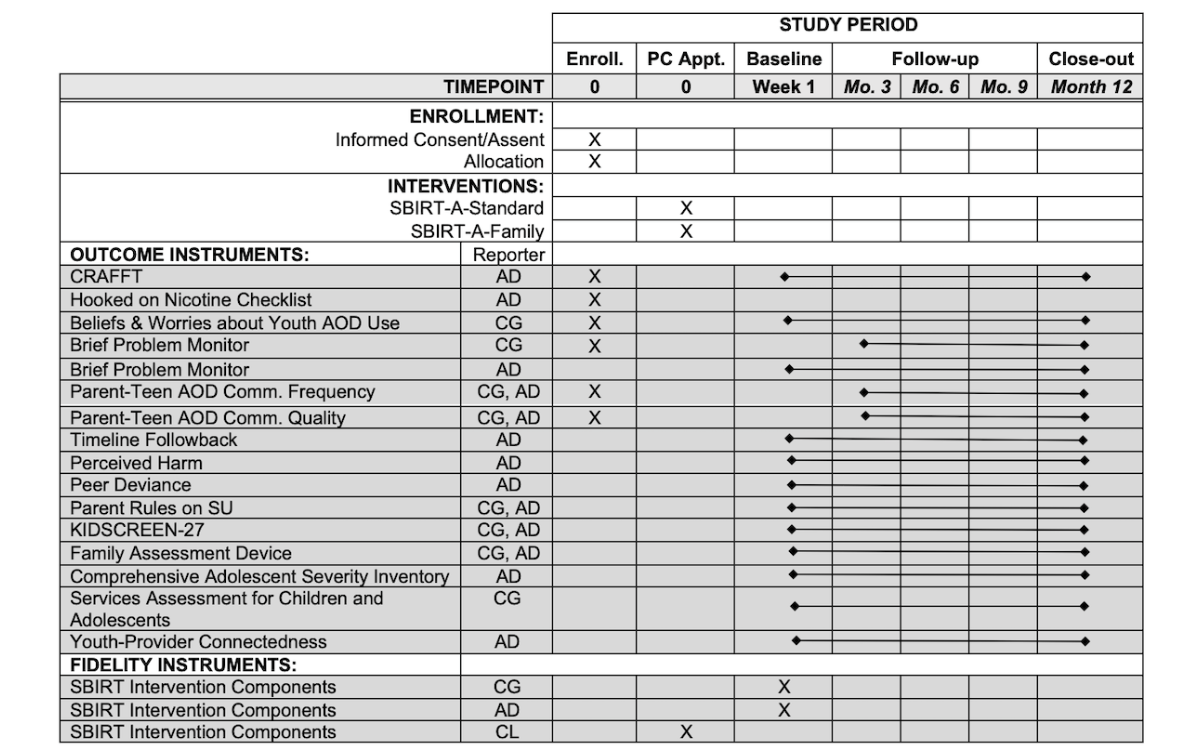
The SPIRIT (Standard Protocol Items: Recommendations for Interventional Trials) checklist for the screening, brief intervention, and referral to treatment for adolescents: standard adolescent-only approach (SBIRT-A-Standard) versus SBIRT-A: family-based approach (SBIRT-A-Family) for adolescent substance use (SU) in the primary care (PC) multisite randomized effectiveness trial: schedule of trial enrollment, interventions, and assessments. AD: adolescent participant; AOD: alcohol and other drug; CG: caregiver participant; CL: clinician participant.

Every adolescent or family assigned to an *at risk* category in either condition is enrolled until study capacity is reached (n=2000). In addition, a randomly selected subsample of adolescents (n=300; balanced across conditions) screened as *low risk* are followed for outcome assessments; the selection of the group categorized as *low risk* uses quota sampling based on sex, race, ethnicity, and age. Outcome data are collected via remote digital assessment methods at initial assessment (as soon as possible after the PC visit) and at 3, 6, 9, and 12 months after the PC visit using REDCap Cloud software (Research Electronic Data Capture developed at Vanderbilt University and licensed to cloud-based technology company nPhase, Inc), a widely used secure, Health Insurance Portability and Accountability Act (HIPAA)–compliant, web-based data collection platform. Figure 2 shows the SPIRIT (Standard Protocol Items: Recommendations for Interventional Trials) checklist depicting the schedule of trial enrollment, interventions, and assessments. All youth and caregivers separately receive both email and SMS text prompts when assessments are due, using contact information provided during the consenting process. When either youth or caregiver fails to complete a follow-up assessment, research staff members blinded to condition assignment reach out to arrange a live assessment via telephone or videoconference. Each assessment requires approximately 20 minutes to complete.

Clinicians and families enrolled in the study retain the right to withdraw consent at any time. The protocol will be discontinued at any site where procedures become burdensome or otherwise impinge on the routine performance of participating staff. Analysis of intervention impacts and potential harm will be continuous throughout the trial. In cooperation with the administration of the partnering sites, investigators will provide full study debriefing and offer counseling referrals to any participant aggrieved or injured due to trial participation.

### Study Conditions

Both study conditions follow a similar set of procedures anchored in the use of handheld tablets. As depicted in Figure 1, tablets are used to collect screening data from youth and caregivers, calculate youth and family risk levels, and offer digitally supported BI and RT activities to users. Point-of-care digital SBIRT procedures can improve patient outcomes by facilitating more rapid and standardized services, data-informed clinical decision-making, and improved practice efficiency [16,51]. Both conditions feature 2 types of digital BIs: *patient facing* (ie, self-administered: patients navigate the BI alone on the tablet) and *provider facing* (ie, provider administered: providers and patients jointly navigate the BI on the tablet). The total time needed to deliver each approach matches the typical wait time for preventive visits plus time devoted to delivering comparable SBIRT-A procedures for youth identified as at risk in routine PC. Both conditions allow all youth and caregivers to speak with providers about AOD issues at any point they choose, in keeping with best practices honored at partner sites. When study interventions are not delivered during the main visit, providers can opt to complete interventions in a follow-up visit scheduled within 1 week or complete immediate handoff to an on-site behavioral health specialist (trained in all study procedures) to complete the intervention [21,52]. In addition, caregivers in both conditions are digitally prompted to enroll in a web-based suite of caregiver-focused resources (helpline, parent coaching, parent groups and electronic resources, and SMS text messaging) specifically designed to support youth AOD risk reduction and caregiver self-care [53].

### SBIRT-A-Standard Procedures

#### Screening (Approximately 2 Min)

All consented youth aged 12 to 17 years complete the CRAFFT (car, relax, alone, forget, friends, trouble) screening tool [54,55], which assesses the number of days during the past year and then the past 3 months during which various formulations of AOD were used, as well as whether they have ridden in a car whose driver was intoxicated; youth who respond negatively to all questions are categorized as *low risk*. If youth report >0 days of AOD use, the tool asks 5 additional questions assessing use risk and consequences; in addition, youth who report nicotine use in the past 30 days complete a nicotine dependence measure, the Hooked on Nicotine Checklist [56], modified to include nicotine vaping. Screening data are then used to sort youth into 3 risk categories: *riding risk only* (no reported AOD use but indicated rode in car driven by intoxicated person), *distant use* (reported AOD use in past year but not past 3 months), or *recent use* (reported AOD use in past 3 months). All screening data are summarized in a tablet-delivered clinician report to guide provider-facing intervention activities.

#### AOD Psychoeducation: All Risk Categories (Approximately 5 Min)

In the wait area, youth receive a tablet-delivered brief digital AOD education tutorial that includes advice to abstain from, or reduce, AOD use. The tutorial focuses on adolescent AOD prevalence rates and related behavioral symptoms, AOD use neurobiology and its relation to adolescent health, and common AOD impacts on developmental milestones [57]. Psychoeducation for AOD has shown positive effects as both a universal and selective prevention strategy [58,59].

#### Brief Negotiated Interview: Distant Use (Approximately 5 Min) and Recent Use (Approximately 8 Min)

In PC offices, youth and providers together complete a tablet-supported brief negotiated interview (BNI) [60]. The BNI is informed by AOD use data gathered during youth screening [61]. The BNI focuses on (1) education about AOD disorders, including youth and family factors that impact AOD use; (2) user-tailored feedback comparing the given youth’s AOD use and related problems to national norms, along with information on the neurobiological effects and developmental impacts of frequent use [32]; (3) motivational tools (eg, reduction readiness rulers) and decisional balance exercises (weighing positive vs negative personal impacts of AOD use) tailored to the youth’s use levels [62,63]; and (4) AOD reduction goal-setting interventions tailored to the youth’s readiness to change AOD use [64]. BNIs of this kind have demonstrated effectiveness in reducing AOD use and related problems [14,32,65].

#### RT: Recent Use (Approximately 5 Min)

In PC offices, providers and youth discuss the value of attending counseling services to address AOD-related problems [66] and counseling referral links that the PC site curates with local services, as well as the value of youth talking directly with caregivers about their AOD involvement as a first step toward support seeking and behavior change [45]. Providers directly recommend AOD counseling and facilitate a first appointment for youth who agree. The study team will continue to monitor and update curated referral lists with availability and wait-list times throughout the duration of the study.

### SBIRT-A-Family Procedures

#### Screening (Approximately 2 Min Apiece)

Screening procedures incorporate CRAFFT procedures for youth described for the SBIRT-A-Standard condition. They also incorporate two sources of caregiver-report data: (1) an assessment of caregiver beliefs about their child’s AOD use and worries about the impact of AOD use, and (2) a well-validated checklist of youth mental health problems [67] that yields clinical cut scores for 4 domains. If both youth and caregiver report no AOD use or clinically elevated mental health problem, the family is categorized as *low risk*. Otherwise, screening data are combined to sort families into 3 risk categories: *hidden substance use risk* (youth reports AOD use in past year; caregiver reports no youth AOD use in past year), *named mental health risk* (caregiver reports no youth AOD use in past year; caregiver reports clinical-level score for at least 1 youth mental health domain), and *named substance use risk* (caregiver reports youth AOD use in past year with or without reporting a clinically elevated mental health score). Adolescent and caregiver screening data are both summarized in the clinician report to guide provider-facing intervention activities.

#### AOD Psychoeducation: All Risk Categories (Approximately 5 Min Apiece)

Youth proceed as indicated in the SBIRT-A-Standard condition based on youth screening data. In the wait area, caregivers receive a tablet-delivered parenting tutorial that covers two AOD risk domains: (1) education about adolescent AOD use, including prevalence rates, related behavior problems, neurobiological and health effects, and impacts on developmental milestones; and (2) education and video modeling about parenting strategies that reduce or moderate AOD use risk, including positive communication, fair and consistent discipline, and nonjudgmental conversations about AOD use [68]. Positive parenting education has been shown effective as a universal and selective prevention strategy in parent-focused AOD prevention trials [69-71]. The tutorials are tailored for each risk category.

#### BNI: Named Substance Use Risk (Approximately 5 Min Apiece)

Youth proceed as indicated in the SBIRT-A-Standard condition based on youth screening data. In PC offices, caregivers and providers together complete a tablet-supported parenting BNI informed by data from the caregiver screening only that parallels the youth BNI (eg, AOD education and developmental risks) and also includes motivation, modeling, and goal setting on positive parenting (eg, nonjudgmental conversations) and effective parent-youth communication about AOD use (Multimedia Appendix 3) [41].

#### Facilitated Conversation: Named Substance Use Risk (Approximately 5 Min)

In PC offices, providers meet with youth and caregivers together to discuss the value of talking directly with the other family member about AOD risk. Providers convene a brief facilitated conversation coaching youth and then caregivers to speak directly to one another about AOD risk using positive communication skills presented to caregivers via wait area psychoeducation. In this conversation providers (1) emphasize that a positive youth-caregiver relationship is the strongest protective factor for youth development and (2) follow guidelines for brief triadic risk reduction interventions focused on positive family communication about AOD risk (Multimedia Appendix 4) [72]. This facilitated conversation is meant to follow the caregiver BNI. Providers may conduct the youth BNI (when indicated) before the caregiver BNI or after the facilitated conversation depending on preference and workflow.

#### RT: Named Substance Use Risk (Approximately 5 Min)

When indicated, providers directly recommend AOD counseling and facilitate a first appointment for families who agree (Multimedia Appendix 5).

### Intervention Training and Fidelity Monitoring

Site PC clinic staff who provide SBIRT-A to clinic patients are trained by experts in all intervention procedures for both conditions. We expect to train 10 providers per clinic. Providers attend a 1-hour site orientation followed by 3 hours of live training. First, providers complete a 1-hour introduction to motivational interviewing for adolescents, caregivers, and families. Next, providers attend two 1-hour live training sessions to apply motivational interviewing principles to support adolescent, caregiver, and family behavior change. The self-study training will include video-recorded presentations on motivational interviewing principles. Live training will include a review of counseling steps for each intervention, video-recorded counseling demonstrations, and opportunities to practice counseling steps. These training procedures have been successfully implemented in similar PC SBIRT studies [16,31]. Baseline fidelity is established via a 1-hour individual counseling practice with research staff. Thereafter, providers attend a monthly 1-hour clinical consultation meeting held by authors MB and NPP to maintain and enhance intervention skills, review notable clinical encounters, and troubleshoot procedural and technology challenges. In addition, providers will complete practice interventions using standardized case vignettes on a 6-month cadence. Vignette scenarios will be informed by implementation experiences shared by providers during the course of the consultation period; these serve as training refreshers and will also provide fidelity feedback data to be incorporated in consultation meetings, as will data summarized from the provider self-report fidelity checklist described in the next subsection [73].

### Fidelity Measures

A provider self-report fidelity checklist is collected after every encounter with every family. The checklist assesses information on which the provider-facing intervention components were delivered: adolescent BNI, caregiver BNI, facilitated conversation, or RT. Checklist items feature 3-point Likert-type rating scales to indicate how clinically helpful the given component was for the encounter: *not at all*, *somewhat*, or *very*. The checklist also logs (1) which implementation scenario was selected for intervention delivery: pediatrician intervention during visit, pediatrician follow-up visit, or behavioral specialist intervention on the visit day; (2) whether the intervention occurred in person or via video call; and (3) the number of minutes spent delivering the intervention or interventions. Research indicates that clinicians can reliably self-report on the delivery of behavioral interventions with families using brief postencounter fidelity checklists [74-76]. In addition, during initial follow-up assessment, adolescents and caregivers report on which components they experienced during the encounter: psychoeducation, BNI, facilitated conversation, or RT.

### Outcome Measures: Screening Interview

CRAFFT [54,55] is a widely used and well-validated youth report tool that measures patient use of alcohol, cannabis, nicotine, illegal drugs, prescription medication, or anything else to get high in the past year and the past 3 months. It also asks about riding in a car driven by someone (including self) who was intoxicated (described in the SBIRT-A-Standard Procedures subsection). If AOD use is reported, the tool asks 5 additional questions: “Do you ever use alcohol or drugs to relax, feel better about yourself, or fit in?” “Do you ever use alcohol or drugs while you are by yourself [alone]?” “Do you ever forget things you did while using alcohol or drugs?” “Do your family or friends ever tell you that you should cut down on your drinking or drug use?” “Have you ever got into trouble while you were using alcohol or drugs?” The Hooked on Nicotine Checklist [56] is a 10-item youth report of nicotine dependence completed by patients who report any days of using a vaping device containing nicotine, or any tobacco products, during the past 30 days. Parent-Adolescent AOD Communication Frequency [77] is a 6-item youth and caregiver report of how often parents and teens talk to one another about key AOD issues (eg, the health risks of use and discipline regarding use) scored on a 5-point scale ranging from *never* to *very often* [78-80]. Parent-Adolescent AOD Communication Quality [81] is a 6-item youth and caregiver report of the quality of communication between parent and teen about AOD issues (eg, “My caregiver/teen and I are interested in each other’s opinions about AOD” and “If my caregiver/teen and I talk about drugs, I feel understood”) scored on a 5-point scale ranging from *not at all* to *very much* [41,77]. Beliefs and Worries about Youth AOD Use is an 8-item measure that asks caregivers what they believe about their teen’s use of AOD in the past year and in the past 3 months (response options: “I am sure or pretty sure my teen has not used,” “I suspect but I am not sure my teen has used,” and “I am sure or pretty sure my teen has used”). Follow-up questions ask which substances they believe their teen has or may have used and their worries about the impact of their teen’s use of AOD across contexts (ie, home, school, peers, and safety). This measure was created for this study, inspired by the Screening to Brief Intervention tool [8] and the CRAFFT tool [55] and vetted by SBIRT experts. The Brief Problem Monitor is a 19-item caregiver report component of the well-validated Achenbach youth behavior problem assessment system [67] that yields normed scores with clinical cut levels for 4 problem domains: internalizing (anxiety, depression, and somatic complaints), externalizing (aggression and conduct problems), inattention or impulsivity, and total problems [82].

### Outcome Measures: Follow-Up Interviews

The timeline followback (TLFB) method [83] is the gold standard method for collecting youth report retrospective estimates of AOD use using a calendar visual aid to facilitate recall. This study used a computer-administered and calendar-assisted instrument created for REDCap-compatible remote asynchronous follow-up assessment modeled after the TLFB [31]. This version assesses the number of days that youth used alcohol, tobacco, marijuana, non–medical prescription drugs, inhalants or vapes, and herbs or synthetic drugs over the past 90 days. If alcohol was used, follow-up questions assess binge drinking episodes. The KIDSCREEN-27 questionnaire (youth and caregiver report) [84] measures youth functioning in social and school domains. Items assess the degree to which youth have experienced problems with a specific task or activity during the past month. It is internationally used and has excellent psychometric properties with strong construct validity and test-retest reliability [84,85]. The Services Assessment for Children and Adolescents (caregiver report) [86] assesses the youth’s past and current use of inpatient, outpatient, and school-based behavioral health services. It has shown strong validity and test-retest reliability [87] as well as interrater reliability between parent and child reports [88]. Items were added to assess caregiver enrollment and the use of web-based educational and support resources for adolescent AOD. Perceived Risk (youth report) [89] asks youth to rate the degree to which people risk harming themselves if they use various categories of substances on a 4-point scale ranging from *no risk* to *great risk*. Peer Deviance (youth report) [89] assesses the number of peers who use various substances on a 5-point scale ranging from *none* to *all*. Parent Rules about Substance Use (youth and caregiver report) [90] assesses parent rules about using various substances on a 6-point scale ranging from *not allowed to use AOD* to *no rules about using AOD*. The Family Assessment Device (youth and caregiver report) [91] assesses family relationships, communication, and problem-solving on a 4-point scale ranging from *strongly agree* to *strongly disagree*. The Comprehensive Adolescent Severity Inventory (youth report) [92] assesses academic achievement, school connection, and disciplinary record. Youth-Provider Connectedness (youth report) [93] assesses the level of trust and connection adolescents feel toward their health care providers on a 5-point scale ranging from *not at all* to *very much*. Psychoeducation Knowledge (caregiver report; created for this study) assesses caregiver knowledge of the tablet-delivered AOD psychoeducation. The Parent-Adolescent AOD Communication Frequency measure, Parent-Adolescent AOD Communication Quality measure, and Brief Problem Monitor (all described in the previous subsection) are also administered at follow-up to youth and caregivers.

### Plan of Analyses

#### Aim 1 Analyses

Aim 1 evaluates the effectiveness of SBIRT-A-Family compared to SBIRT-A-Standard in preventing the escalation of AOD use. AOD use is measured using the TLFB, which computes the percentage of the days of use over the past 90 days across a range of substances. Given that we expect the percentage of the days of AOD use to be highly skewed within this population identified as predominantly low risk, we will collapse outcomes on this measure onto a 4-point ordinal scale indicating the levels of AOD use as follows: *none* (0% days of use), *once per month* *or less* (1%-3% days of use), *between once per month and once per week* (4%-14% days of use), and *once per week or more* (≥15% days of use). Aim 1 hypotheses are tested using a mixed effects logistic regression model for ordinal longitudinal outcomes [94]:



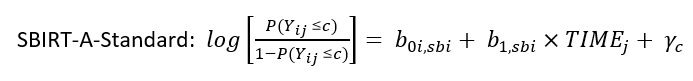

**(1)**




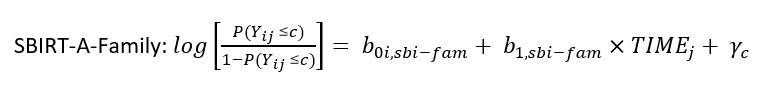

**(2)**


wherein (1) and (2) are jointly estimated submodels for the SBIRT-A-Standard and SBIRT-A-Family conditions, respectively. The dependent variable in each equation is the log odds (*logit*) that AOD use response *Y* for person *i* at time point *j* (*j*=1 to 5) is in category *c* (*c*=1 to *C*−1) or lower where *c* is one of the ordinal response categories created from the AOD measure. The *b*_0_*_i_* parameters in (1) and (2) are random intercepts estimating the log odds of *Y_ij_*≤*c* at initial assessment in each condition; the *b*_1_ parameters estimate the linear rate of change (ie, slope) in the log odds of *Y_ij_*≤*c* across measurement points, which are indexed by the variable *TIME_j_*. The *γ_c_* parameter estimates thresholds between adjacent categories on the given AOD variable. The intercept in each submodel is specified as a random effect to account for the nesting of repeated measurements within individuals. Slope parameters are specified as fixed effects. The model is specified as jointly estimated simultaneous equations for each study condition, rather than a single model containing an intervention main effect and interaction term, to allow for greater flexibility in modeling group-specific effects as well as for comparing each condition to the normative comparison (NC) group of youth (in which the given youth, standard condition or youth and family, family condition, screened negative). Changes in the levels of AOD use are captured by the *b*_1_ parameters in each submodel, where positive values of *b*_1_ indicate an increase in the log odds of being in a lower AOD use category on each logit over time compared to the initial assessment. The causal effect of assignment to SBIRT-A-Standard versus SBIRT-A-Family on AOD use (ie, the intent-to-treat effect) is estimated as the difference between *b*_1_*_,sbi−fam_* and *b*_1_*_,sbi_*_._ Separate models are fitted for each AOD use outcome variable. To account for nonindependence due to the clustering of individuals within clinic sites, indicator variables controlling for the fixed effect of each clinic will be included as covariates in submodels (1) and (2) [95].

#### Evaluating Clinical Significance

The clinical significance of intervention effects on youth AOD use at 1-year follow-up is evaluated by gauging the degree to which youth identified as at risk resemble youth in the NC group, which provides a normative benchmark against which to compare changes in AOD use in the 2 study conditions. Analyses are performed by adding a third submodel to those shown in (1) and (2) that estimates change in AOD use levels between initial assessment and 1-year follow-up among NC youth. By definition, 100% of NC youth are in the *none* AOD use category at initial assessment. On the basis of preliminary analyses using data from the NSDUH, this percentage is expected to decline to approximately 90% at 1-year follow-up, whereas increases are expected in both study conditions. The difference in the likelihood (ie, log odds) of reporting AOD use in the *none* category at 1-year follow-up in each study condition compared to the NC group is quantified in the following equations:

NC versus SBIRT-A-Standard: [(*b*_0_*_i_*_,_*_no−risk_* + *b*_1,_*_no−risk_* × 4 + *γ*_1_) – (*b*_0_*_i_*_,_*_sbi_* + *b*_1,_*_sbi_* × 4 + *γ*_1_)] **(3)**

NC versus SBIRT-A-Family: [(*b*_0_*_i_*_,_*_no−risk_* + *b*_1,_*_no−risk_* × 4 + *γ*_1_) – (*b*_0_*_i_*_,_*_sbi−fam_* + *b*_1,_*_sbi−fam_* × 4 + *γ*_1_)] **(4)**

where *b*_0_*_i_*_,_*_no−risk_* and *b*_1,_*_no−risk_* are the intercept and slope parameters in the mixed effects longitudinal logistic regression submodel for the group, *4* is the coded value for *TIME_j_* at 1-year follow-up, and *γ*_1_ is the threshold for the *none* category on AOD use variable *Y*. A log odds value of 0 resulting from the aforementioned differences indicates equivalence between the groups, whereas positive log odds values indicate a higher likelihood of reporting *none* at 1-year follow-up in the NC group. These differences are converted into odds ratios and then into Cohen *d* values using an “odds ratio to *d*” conversion formula [96]. The Cohen *d* value between the NC and SBIRT-A-Family groups is expected to be in the *small* range (0.10-0.30), and the Cohen *d* value for the NC versus SBIRT-A-Standard comparison is expected to be in the *moderate* range (0.40-0.60). Small effect size values would indicate high concordance in AOD use between the NC group and the at-risk study categories, thereby supporting the clinical significance of intervention effects at 1-year follow-up.

#### Aim 2 and Aim 3 Analyses

Aim 2 evaluates the comparative effectiveness of study conditions on AOD risk factors (eg, peer conduct problems, susceptibility to peer pressure, school problems, and family functioning), co-occurring problems (internalizing, externalizing, and impulsivity), quality of life, and parent-adolescent AOD communication. A similar modeling approach to aim 1 is used, the primary difference being that all outcome variables are continuous rather than ordinal; as such, standard mixed effects growth models [97] are used to compare intervention effectiveness. Aim 3 compares the rates of behavioral counseling service use between the conditions. Differences are tested at 1-year follow-up using a simple logistic regression model with a binary *yes/no* outcome variable indicating whether the youth received any counseling during the study period. Higher rates of counseling use are expected in the SBIRT-A-Family condition.

#### Aim 4 Analyses

Aim 4 examines comparative effectiveness on AOD use outcomes within the subgroups of interest: age, sex, race, ethnicity, and youth AOD risk category (*recent use*, *distant use*, *ride risk only*, and *low risk*). To examine differential intervention effectiveness based on youth age, sex, and race or ethnicity, these demographic predictors are added to the submodels for aim 1 as exploratory analyses; there are no hypotheses for interactive effects. To examine differential intervention effectiveness based on risk category, analyses will be conducted separately for SBIRT-A-Standard and SBIRT-A-Family; the respective risk categories are added to the submodels for aim 1 as exploratory analyses for each condition, again with no specific hypotheses for interactive effects.

#### Missing Data and Multiple Significance Tests

Missing data are addressed using multiple imputation, a Bayesian procedure that simulates missing values in a data set based on the nonmissing data values and a prior distribution of the missing values, given the nonmissing data [98]. Multiple imputation yields valid results under the assumption that data are missing completely at random or missing at random, given a set of measured covariates. Potential violations of random data missingness due to study protocol dropout can be mitigated by incorporating baseline variables predicting dropout status into multiple imputation models [99]. A set of baseline variables is identified to predict dropout status in each condition. Baseline predictors of dropout are included as covariates in the models to control for potential nonrandomly missing data; the imputation procedure is run separately in each condition.

To control type I error inflation due to multiple tests of statistical significance while also conserving power [100], the false discovery rate procedures developed by Benjamini and Hochberg [101] are used. False discovery rate correction adjusts *P* values based on the number of *P* values <.05 across all tests; as such, it provides a more tolerable balance between type I and type II error rates.

### Study Power

Projecting 2000 youth identified as at risk enrolled in the study and allowing for a 20% attrition rate, a final 1-year follow-up sample size of 1600 youth identified as at risk is anticipated. Statistical power to detect small intervention effects on alcohol use (aim 1) was estimated; to be conservative, multiply imputed data for attritted cases were not included in the power analyses. An a priori power analysis was conducted using a Monte Carlo simulation [102]. Specifically, 1000 simulated data sets of 1600 youth randomly assigned to condition were generated. Each youth in each simulated data set had 5 follow-up outcome measurements (initial assessment and at 3, 6, 9, and 12 months), and youth were nested in 3 sites in proportions equal to those reported by PC partner sites. The intervention effect size was simulated as an increase in the proportion of youth reporting alcohol use in the *none* category at each assessment. On the basis of the preliminary analyses of data from the 2023 Monitoring the Future national survey [1], it was projected that 62.3% of the youth identified as at risk would report alcohol use in the *never* category at initial assessment, increasing to 78.4% in the SBIRT-A-Family condition and 67.9% in the SBIRT-A-Standard condition at 1-year follow-up. These increases represent a small effect size difference compared to the NC group at 1-year follow-up for SBIRT-A-Family, a moderate effect size difference compared to the NC group at 1-year follow-up for SBIRT-A-Standard, and a small effect size difference at 1-year follow-up between the 2 at-risk categories. The simulation program assumed a correlation of 0.90 between repeated measures within individuals. In addition, on the basis of power calculations in a similar randomized trial by Sterling et al [15], an interclass correlation of 0.02 was assumed to reflect the within-site clustering effect. To estimate power to detect an intervention effect on alcohol use, the submodels in (1) and (2) were fit to all 1000 simulated data sets. In each simulated analysis, the significance of the difference in the slope parameters between the submodels (*b*_1,_*_sbi−fam_*−*b*_1,_*_sbi_*) was tested. Power was computed as the proportion of *P* values <.05 across the 1000 sets of results, which was 0.99. Thus, the trial is well powered to detect small intervention effects on alcohol use. A similar level of power to detect effects on other substances is assumed, given that these outcomes are measured using the same scale. Power on aim 2 and aim 3 outcomes is expected to be >0.95, given that these outcomes are measured on continuous scales. The study is also powered for analyses aimed at examining moderator effects for demographic subgroups [21] and risk categories (aim 4); for example, to estimate power to detect effects within youth AOD risk categories, power analyses were repeated within the subsample of simulated youth classified as *recent use*, projected to comprise approximately 36% of the full sample based on the preliminary analyses of data from the 2020 NSDUH [103]. A power of 0.95 to detect a small effect on the change in the proportion of youth reporting alcohol use in the *never* category among youth categorized as *recent use* was obtained. These results extend to any subsample that comprises at least 35% (560/1600) of the projected sample; subsamples of at least this size are projected for male and female participants; Black, Hispanic, and White non-Hispanic youth; and younger and older youth.

### Ethical Considerations

Central ethics approval for all study activities has been obtained from Solutions IRB (2023/01/3) and local ethics approval from research sites has been obtained. Caregivers and youth will independently provide informed consent or assent before the initiation of study activities (Multimedia Appendix 6). All study data will be collected on encrypted and password-protected tablets and stored on REDCap Cloud, a HIPAA-compliant data platform. All study activities will be subject to monitoring by the data safety and monitoring board of the same institution (Multimedia Appendix 7). Any modifications to the protocol that might impact the conduct of the study or its specified objectives and procedures will require a formal amendment to the protocol and approval by the institutional review board and the data safety and monitoring board before implementation. Families will receive a US $10 Amazon gift card for the completion of the screening, and each participant will receive US $170 in gift card vouchers of their choice during the follow-up period delivered via Tango Card (US $30 each for the initial assessment and 3- and 6-month follow-ups and US $40 each for the 9- and 12-month follow-ups).

## Results

This study will be conducted over a 5-year period. Provider training was initiated in year 1 (December 2023). Participant recruitment and follow-up data collection began in year 2 (March 2024). We expect the results from this study to be published in early 2027.

## Discussion

### Summary

**Figure 3:**
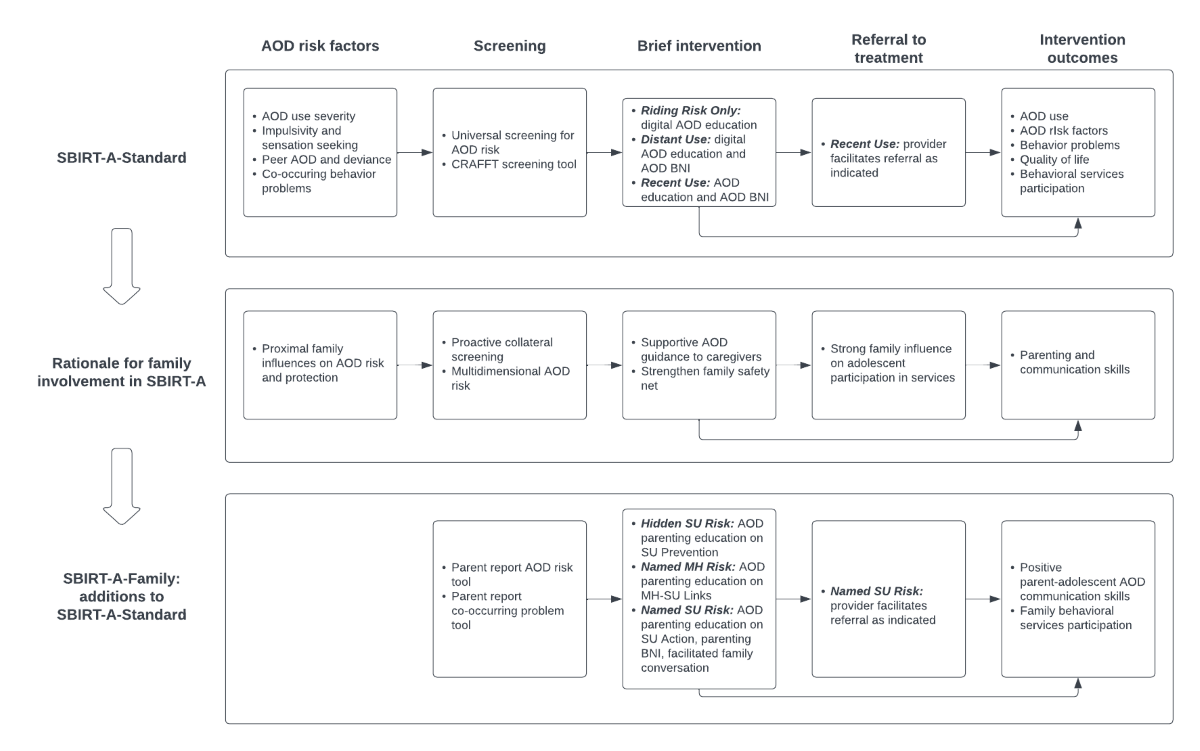
Conceptual framework for the screening, brief intervention, and referral to treatment for adolescents: standard adolescent-only approach (SBIRT-A-Standard) versus SBIRT-A: family-based approach (SBIRT-A-Family) for adolescent substance use (SU) in the primary care multisite randomized effectiveness trial (family-based additions to SBIRT-A for adolescent alcohol and drug use in primary care). AOD: alcohol and other drug; BNI: brief negotiated interview; CRAFFT: car, relax, alone, forget, friends, trouble; MH: mental health.

SBIRT-A is widely endorsed but currently underused in pediatric PC settings, and urgent questions remain about optimal approaches and overall effectiveness [21,104]. In particular, RT procedures in PC remain virtually untested among youth [66]. In addition, whereas research strongly supports involving families in various kinds of interventions for adolescent AOD [17], SBIRT-A effectiveness trials testing approaches that actively engage family members in PC are absent. This trial is designed to help fill these research gaps. Figure 3 presents a framework that depicts key conceptual and methodological factors across intervention components for the adolescent-only SBIRT-A-Standard approach, specifies the rationale for involving caregivers in all SBIRT-A components, and identifies key additions to the SBIRT-A-Standard approach that are introduced by the innovative SBIRT-A-Family approach. This framework aligns closely with kindred conceptual models for family-based AOD prevention in general [69,71] and for family-focused AOD prevention approaches specifically designed for PC [27,105,106], as well as for family-based SBIRT-A models in PC [43,107].

Although our study is not designed to evaluate implementation feasibility, both approaches tested in this trial feature procedures designed to surmount common barriers to SBIRT-A implementation in PC settings: the use of digital resources to enhance provider knowledge and intervention delivery, the embedding of interventions into routine workflow with minor disruption, ongoing technical assistance that incorporates site-specific data performance indicators, and the like [24,25]. Thus, we will gain important information about potential implementation strategies that we will test in future implementation trials once the most effective SBIRT intervention model is identified [49]. Ultimately, the trial results will inform the critical health decision of whether and how to include caregivers in SBIRT-A activities conducted in pediatric care. Guidelines for delivering SBIRT-A procedures based on the trial results will directly inform PC clinical practice and directly benefit the general pediatric population of adolescents at risk for AOD problems and their caregivers.

Importantly, this trial also emphasizes patient-centered outcomes research methods in several ways. It is recruiting participants representative of the full spectrum of adolescents and caregivers attending PC visits. Both SBIRT-A approaches rigorously protect the privacy and confidentiality of both youth and caregivers; neither learn directly or indirectly about the confidential information collected from the other person on surveys or during provider interviews without expressed permission to share such data. All SBIRT-A procedures can be delivered by trained staff across the PC provider spectrum: nurse practitioners, physicians, physician assistants, behavioral counselors, and so forth [108]. These patient-centered design features enhance the consistency and effectiveness of SBIRT-A delivery in PC.

### Study Limitations

As all partner sites are hospital affiliated and located in major urban centers, the study results may not be fully generalizable to pediatric PC practices that operate in distinctly different geographic regions (eg, rural settings), under different health care organizational structures, or without on-site internet support. Due to study inclusion criteria, the results will not be fully generalizable to the population of youth who attend PC visits without caregivers (such youth tend to skew as older) or to youth and caregivers who are not fluent in English or Spanish. The RT procedures embedded in both study conditions are limited to the degree that a given PC site has limited or no options for referring patients to behavioral care providers who serve teenagers with AOD problems. It is beyond the scope of SBIRT-A to intervene directly in cases of multigenerational AOD use, including when the caregiver is experiencing AOD problems; pediatric clinics routinely contend with this matter. Regarding condition allocation, a primary advantage of individual-level over site-level randomization is higher statistical power [109]. The primary drawback of individual-level randomization is the potential for cross-contamination between study conditions, which could dampen intervention effect sizes and reduce statistical power [110]. Cross-contamination is not expected to be substantial in this trial, given that both conditions feature large subgroups of patients who will receive SBIRT-A content in strictly digital format (ie, no research interaction with providers).

## Appendix

Multimedia Appendix 1 The SPIRIT (Standard Protocol Items: Recommendations for Interventional Trials) 2013 checklist: recommended items to address in a clinical trial protocol and related documents.

Multimedia Appendix 2 Study conditions of the screening, brief intervention, and referral to treatment for adolescents: standard adolescent-only approach (SBIRT-A-Standard) versus SBIRT-A: family-based approach (SBIRT-A-Family) for adolescent substance use in the primary care multisite randomized effectiveness trial.

Multimedia Appendix 3 Caregiver brief negotiated interview.

Multimedia Appendix 4 Family-facilitated conversation.

Multimedia Appendix 5 Family referral to treatment.

Multimedia Appendix 6 Screening, brief intervention, and referral to treatment for adolescents adult consent form.

Multimedia Appendix 7 Study committees and teams.

Multimedia Appendix 8 Peer-review report by the Patient-Centered Outcomes Research Institute.

## Abbreviations

AOD: alcohol and other drug
BI: brief intervention
BNI: brief negotiated interview
CRAFFT: car, relax, alone, forget, friends, trouble
HIPAA: Health Insurance Portability and Accountability Act
NC: normative comparison
NSDUH: National Survey on Drug Use and Health
PC: primary care
RT: referral to treatment
SBIRT: screening, brief intervention, and referral to treatment
SBIRT-A: screening, brief intervention, and referral to treatment for adolescents
SPIRIT: Standard Protocol Items: Recommendations for Interventional Trials
TLFB: timeline followback

## Data Availability

The data sets generated and analyzed during this study will be archived on the web via the National Institute of Mental Health Data Archive.
