# Supplementary material for: Standard Versus Family-Based Screening, Brief Intervention, and Referral to Treatment for Adolescent Substance Use in Primary Care: Protocol for a Multisite Randomized Effectiveness Trial
Source: JMIR Res Protoc. 2024 May 31;13:e54486. doi: 10.2196/54486 (PMC11179044; doi:10.2196/54486)
Supplement: Multimedia Appendix 2 [file resprot_v13i1e54486_app2.pdf]

SBIRT-A-STANDARD	SBIRT-A-FAMILY
**SCREENING**	
**(~2 mins; wait area)**<br>• Adolescent completes CRAFFT screen tool<br>• Alcohol, cannabis, other drug use during past year and 3 months<br>• Nicotine use past year and 30 days<br>• Adolescent assigned a Risk Category: Low Risk, Riding Risk Only, Distant Use, Recent Use	**(~2 mins apiece; wait area)**<br>• Adolescent screen procedures, per SBIRT-A-Standard condition<br>• Caregiver completes AOD risk screen tool<br>• Beliefs about adolescent AOD use<br>• Adolescent MH symptoms: internalizing, externalizing, attention<br>• Family assigned a Risk Category: Low Risk, Hidden SU Risk, Named MH Risk, Named SU Risk
**PSYCHOEDUCATION**	
**Riding Risk Only, Distant Use, Recent Use (~5 mins; wait area)**<br>• Adolescent receives patient-facing digital AOD psychoeducation:<br>• AOD prevalence rates<br>• AOD use neurobiology and risks<br>• AOD impacts on development	**Hidden SU Risk, Named MH Risk, Named SU Risk (~5 mins [apiece]; wait area)**<br>• Adolescent proceeds per SBIRT-A-Standard condition<br>• Caregiver receives patient-facing digital AOD parenting education (tailored to Category):<br>• AOD prevalence & behavioral risk<br>• Parenting strategies to reduce risk<br>• Modeling productive adolescent-caregiver conversations about AOD
**BRIEF NEGOTIATED INTERVIEW**	
**Distant Use, (~5 mins; with provider), Recent Use (~8 mins; with provider)**<br>• Adolescent receives provider-facing BNI informed by adolescent screen data:<br>• Feedback comparing adolescent AOD use to national norms<br>• AOD motivation and decision-making	**Named SU Risk (~5 mins [apiece]; with provider)**<br>• Adolescent proceeds per SBIRT-A-Standard condition<br>• Caregiver receives provider-facing BNI informed by caregiver screen data:<br>• Parenting motivation and positive strategies regarding adolescent AOD use

• Goal-setting for continued non-use or use reduction	• Goal setting and modeling for adolescent-caregiver communication about AOD
**FACILITATED CONVERSATION**	
**N/A**	**Named SU Risk (~5 mins; with provider)**<br>• Provider meets separately with adolescent and caregiver to prepare for family conversation<br>• Provider facilitates a family conversation focused on positive AOD communication
**REFERRAL TO TREATMENT**	
**Recent Use (~5 mins; with provider)**<br>• Provider and adolescent discuss AOD counseling needs and referral options	**Named SU Risk (~5 mins; with provider)**<br>• When indicated, provider and family discuss AOD counseling needs and referral options
