# Supplementary material for: Standard Versus Family-Based Screening, Brief Intervention, and Referral to Treatment for Adolescent Substance Use in Primary Care: Protocol for a Multisite Randomized Effectiveness Trial
Source: JMIR Res Protoc. 2024 May 31;13:e54486. doi: 10.2196/54486 (PMC11179044; doi:10.2196/54486)
Supplement: Multimedia Appendix 3 [file resprot_v13i1e54486_app3.pdf]

Caregiver Brief Negotiated Interview

Counseling Step	Counseling Dialogue
**1. Build Rapport**<br>• Ask permission to discuss SU<br>• Ensure privacy of interview	"*Thank you for answering the questions about [Youth's] SU in the waiting room*"<br><br>Ask **permission**: "*Would it be okay if we talked about the information you shared*?"
**2. Review Screening Responses, Specify Concern, Assess Further**<br>• Briefly summarize caregiver SU screen<br>• Elicit specific concerns<br>• Assess current SU communication<br>• Normalize the topic as anticipatory guidance<br>• Ask permission to offer a strategy	"*In the information you provided in the waiting room, you indicated that you think [Youth] may be using [**named substance(s)**], is that right*?"<br><br>"*You also indicated that you're worried about [**named worries**], is that right? What concerns you most right now*?"<br><br>"*Do you sometimes **talk to [Youth] about SU***?"<br>• *"How does it go?"*<br><br>*"I **recommend to all caregivers of teenagers that they hold regular conversations about SU**; even if difficult, those conversations can be very helpful in many ways. Can I help you think about how to talk to [Youth] about [SU concern]?"*
**3. Offer a Communication Strategy**<br>• Emphasize value of open communication<br>• Elicit response	*"It's healthy for families whenever possible to have what we call an **open conversation**. What I mean by open conversation is talking in a way that is **calm, curious, and caring**.*<br><br>***"Open conversations** can help you and [Youth] listen better to each other. This will also help [Youth] be more likely to share their thoughts, feelings, and experiences."*<br><br>*"**What do you think** about this? Does it sound familiar to videos you just watched in the waiting room?"*
**4. Decisional Balance**<br>• Elicit reasons for having open SU conversations (pros)<br>• Elicit reasons why they may not want	*"What are the main **reasons for having open conversations** with [Youth] about SU [concern]?"*<br>• Common pros: educate teen, keep teen safe, effective communication, reduce SU, improve family life<br><br>*"What are the main **reasons for NOT having this type of open conversation** with [Youth] about SU [concern]*?"

open conversations (cons)<br>• Use double-sided reflection	• Common cons: explosive topic, teen argue/sullen/silent, won't help, SU non-negotiable<br><br>*"So on the one hand, you **[pros]**...and on the other hand, you **[cons]**"*
**5. Readiness Ruler**<br>• Assess readiness to change (0-10)	*"Now, thinking about what we've discussed, on a **scale of 0-10**, with 0 being not at all ready and 10 being completely ready, **how ready** are you to have an open conversation with [Youth] about SU [concern]?"*<br><br>• If **1-10**: "*How did you come to choose (#) rather than a lower number, like (X))?"*<br>• If **0:** "*What might need to happen for you to consider having an open conversation with [Youth] about SU [concern]?*
**6. Next Steps (Elicit-Provide-Elicit)**<br>• Reflect change talk<br>• Elicit goals and next steps and anticipate challenges<br>• Elicit response	*"Thinking about what we've discussed, what **steps** are you willing to take to talk with [Youth] about SU [concern]?"*<br>• **Reflect** back change talk<br>• **Affirm** realistic goals<br><br>"*Would it be ok if we set some specific **goals**?*"<br>Sample goals:<br>• More ready: Try an open conversation by Y date<br>• Less ready: Think about how you would start a conversation with your teen about SU [concern]<br><br>*"**What do you think** about what we've discussed?'*
**7. Set Up Facilitated Conversation**<br>• Briefly summarize discussion<br>• Normalize open communication practice<br>• As indicated: Arrange follow-up and/or conclude visit	*"You came up with a **good plan for how you would like to have open conversations** with [Youth] about SU [concern], such as"...*<br><br>*"I ask all my families of teens to practice with me having an open conversation about SU. **I'd like to help you and [Youth] practice today."***<br><br>*"How does that **sound to you**? We'll do it together."*
