# Supplementary material for: Standard Versus Family-Based Screening, Brief Intervention, and Referral to Treatment for Adolescent Substance Use in Primary Care: Protocol for a Multisite Randomized Effectiveness Trial
Source: JMIR Res Protoc. 2024 May 31;13:e54486. doi: 10.2196/54486 (PMC11179044; doi:10.2196/54486)
Supplement: Multimedia Appendix 4 [file resprot_v13i1e54486_app4.pdf]

<u>Family Facilitated Conversation</u>

Counseling Step	Counseling Dialogue
**PRN: Prepare Youth [Alone] for Facilitated Conversation**	• *SU is an important health conversation for teens and their parents. I recommend to all the families that I work with that they practice together here in the office.*<br>• *Are you open to us speaking all together now? The goal is for you to share with [Caregiver}—to have a new kind of conversation about SU issues.*<br>• *Of course you can share anything you'd like to, and I'll support you.*<br>• *Any questions?*
**1. Set Agenda with Caregiver and Youth Together for Facilitated Conversation**	• *SU is an important conversation topic for teens and their parents.*<br>• ***I recommend to all the families that I work with that they practice together here in the office.***<br>• *I'm going to ask you to talk directly to each other now. As much as possible, try to have it be an open conversation. This means being **calm, curious, and caring.***
**2. Invite Youth to Share with Caregiver**	• *[Youth], I'd like you to go first. I'd like you to **share with [Caregiver] something important, a value or a worry, you have related to SU**. What might that be?*<br>• ***Start with "I'd like you to know that…"***<br>• [If Youth is uncertain or non-responsive, share 2-3 suggestions from the list]: *You might say, I'd like you to know that…*
**3. Possible Youth Values/Worries**	• I don't ever want to use substances alone<br>• I sometimes worry about a friend's SU<br>• I know how to keep myself safe around SU<br>• I plan to abstain from SU until I am older<br>• I am worried that SU might get in the way of my goals
**4. Invite Caregiver to Share with Youth**	• *[Caregiver], I'd like you to go next. I'd like you to **share with [Youth] a hope you have related to SU**. What might that be?*<br>• ***Start with "I hope…"***<br>• [If Caregiver is uncertain or non-responsive, share 2-3 suggestions from the list]: *You might say, I*

	hope…
**5. Possible Caregiver Hopes**	• I hope your friends take care of each other around SU<br>• I hope you abstain from SU until you are older<br>• I hope you can speak to me or another adult if you are worried about SU<br>• I hope you are able to access correct information about SU risks and potential harms<br>• I hope there are ways for you to have fun and try new things that don't include SU
**6. Wrap Up**	• *Every teen and every family is trying to figure out how to stay safe and healthy and also stay connected with each other.*<br>• *One way to do that is to have* ***calm, curious, and caring conversations about important topics like SU****. That's why I wanted you to practice here today.*<br>• ***I recommend you continue to have open conversations at home at least once a month.***<br>• *I'm so appreciative of both of you taking the time to speak to me and one another today about your worries and your hopes.*
