# Supplementary material for: Standard Versus Family-Based Screening, Brief Intervention, and Referral to Treatment for Adolescent Substance Use in Primary Care: Protocol for a Multisite Randomized Effectiveness Trial
Source: JMIR Res Protoc. 2024 May 31;13:e54486. doi: 10.2196/54486 (PMC11179044; doi:10.2196/54486)
Supplement: Multimedia Appendix 5 [file resprot_v13i1e54486_app5.pdf]

Family Referral to Treatment

Counseling Step	Counseling Dialogue
**Affirm Strengths**<br>• Set tone for open conversation<br>• Name specific family strengths<br>• Normalize counseling referral	• *Every teen is trying to figure out how to get things done, and also stay connected with friends and family, and work on becoming an adult.*<br>• *During our time together today, we spoke about SU. I saw [insert family strength observed by the provider in the BNI/FC].*<br>• *I know this time of life has a lot of challenges for teens. For many teens and families I see, I recommend getting additional support, in the form of counseling.*
**Recommend Treatment**<br>• Focus on unique rationale for referral and your ideas for how treatment could be helpful<br>• Offer education about counseling	• *I believe counseling could be valuable for you: a private, supportive space for you [Youth] to be heard and talk through some challenges that all teenagers face, and discuss your particular goals.*<br>• *It can also be a place in which you [Youth] learn new skills and get to know yourself better.*<br>• *It's also my hope that you [Caregiver] may also be a part of the counseling so you can continue to support [Youth] like you did today.*
**Be Curious**<br>• Focus on their values related to SU (and mental health if applicable)<br>• Elicit their ideas for what they might gain from counseling	• *What do you think about counseling as a possible next step?*<br>• *What do you think could make counseling as helpful as possible for you [youth] and your family?*
**Develop Plan for Next Steps**<br>• Focus on a concrete course of action for enrollment<br>• Plan for follow up	• *Can I help you think about getting started?*<br>• *The first step is a phone call to schedule an intake.*<br>• *You might have to call a few times, and there might be a waitlist, but I think you should call soon. I recommend you first call [Referral A], and [Referral B] is another good place.*<br>• *Do you have questions for me?*<br>• *I'll contact you in a few days to see how it's going.*<br><br>*OR*<br>• *Are you open to calling now? If you'd like, we can make the call together or I can check back with you in a few minutes.*

**If Family Declines or is Ambivalent: Develop Follow-up Plan**<br>• If family is not interested, schedule follow-up to continue conversation	• *I understand counseling is not something you want to commit to right now. I'd like to follow-up with you both after you've had time to think it over.*<br>• *Can we schedule a time for you to come back in person or for a virtual visit in [name timeframe]?*
