# Supplementary material for: Standard Versus Family-Based Screening, Brief Intervention, and Referral to Treatment for Adolescent Substance Use in Primary Care: Protocol for a Multisite Randomized Effectiveness Trial
Source: JMIR Res Protoc. 2024 May 31;13:e54486. doi: 10.2196/54486 (PMC11179044; doi:10.2196/54486)
Supplement: Multimedia Appendix 6 [file resprot_v13i1e54486_app6.pdf]

SBIRT-A Adult Consent

Introduction

We are asking you to participate in a research study. If you agree to participate, you will need to sign this Informed Consent document. Informed Consent is a written agreement that shows you are willing to participate in this study.

This form will explain:

- the purpose of the study
- what you will be asked to do
- the risks and benefits

It will also explain that you do not need to be in this study to continue getting treatment at this clinic. By signing this form, you do not give up any legal rights. You and your child will still be able to receive the care to which you are entitled.

Why have I been asked to be a part of this study?
The research team is hoping to test two interventions to help address substance use problems among youth. You and your child are being asked to take part in this study because your child is between the ages of 12 and 17 years old, and you are both making a visit to this clinic.

What is involved in this study?
If you agree to participate in this study, you will be asked to (1) provide information on substance use risk factors for your child; (2) complete a short video exercise while you wait for the appointment; (3) complete five follow-up assessments over one year. This information will help us learn more about how to improve healthcare for youth.

Will there be two different groups in this study?
There are two different groups in this study. You will be placed in a study group at random. In Group 1, your child will complete a video exercise related to substance use. In Group 2, both you and your child will complete a video exercise related to substance use. In both groups, your child's doctor will see the information that you and your child provide about substance use risk.

How will the data be used?
Your information will only be shared with the research team. It will be used only for purposes of the research project described in this document. Deidentified data will be added to federally funded data archives to advance scientific research across the field.

Who is responsible for the data collected in this study?
If I have any questions about the study, my participation in this study, or a research-related injury, I may contact the Principal Investigator:

Aaron Hogue, Ph.D.
Partnership to End Addiction, New York, NY 10117
(212) 841-5278; ahogue@centeronaddiction.org

Study Specifics:

Why is this study being done?
The purpose of this study is to improve care for youth at risk of using substances. The study will test new ways to screen for substance use, engage youth and families, and refer them to treatment.

This study is funded by Patient Centered Outcomes Research Institute (PCORI) from March 2023 to February 2028.

How long will I be in this study?
You will be in the study for one year. Today you will be screened (1-6 minutes to be completed in waiting room) and receive a brief intervention (5-10 minutes to take place during appointment). You will then complete five follow-up assessments over one year (each taking 30-40 minutes to complete). Data will be collected via online survey at Baseline (as soon as possible after today's visit) and at 3, 6, 9, and 12 months after this visit. All study activities are expected to take a maximum of 3.5 hours to complete.

How many people will take part in this study?
About 2,300 people will take part in this study. Some of them may be from your clinic.

What are the risks of this study?

There is a small risk to privacy from sharing your personal information. You and your child will always have the option to take a break, check in with the doctor, and/or stop taking part in the research. Doing so will not result in any changes to your treatment.

Are there benefits to taking part in this study?
You may learn new information from videos about youth substance use risk. The information we learn from this study may help other families as well.

Will I receive any payment or other monetary benefits?
You will be paid for completing a brief screen and each of five follow-up assessments. The payment breakdown is as follows: $10 per family at Screen, $30 per assessment at Baseline, 3, and 6 months; $40 per assessment at 9 and 12 months. Payments will be issued as electronic amazon gift cards, delivered by email or text for a maximum amount of $170 across study period.

What other options are there?
You can always choose not to participate in the study. Doing so will not affect the standing of you or your child at the clinic. If you choose to leave the study, you may request that your data be destroyed and/or removed from any study analyses.

What about confidentiality?
We are required to report information about potential abuse of children, or danger to their lives or the lives of others. Otherwise, all information you provide will be kept confidential and will be

used for research purposes only. Identifying information will be collected solely to track participants throughout the study. No identifying information will be attached to study data; all data will be coded with an ID number. All data will be stored in a secure folder on the Partnership to End Addiction's network that can only be accessed by the research team.

All research staff with access to study data are required to complete an online course on the protection of human subjects. They will also be trained to follow federal guidelines for maintaining the privacy of participants throughout the study. The results of the study may be published for scientific purposes but will not include any identifying information. However, any data from your participation in this study may be reviewed by the Patient-Centered Outcomes Research Institute, by any relevant federal agency under the Department of Health, by Solutions IRB (the body that oversees our protection of study participants), or by the persons running this study. Anyone who inspects your records will also be required to keep your identity private.

What are the costs?
There are no costs for you to participate in this study.

What are my rights as a participant?
Taking part in this study is voluntary. You should consent only after you have been given all the necessary information. You may choose not to take part in this study. You are also free to leave at any time. Leaving this study will not result in any penalty or loss of benefits to you or your child.

Informed Consent Approval
I have read this document and I agree to participate in the research study conducted by Aaron Hogue, Ph.D. If, however, I wish to end my participation in this study, I have the right to do so at any time without penalty, even after my data has been collected.

If I have questions about the study or my participation in the study, or would like to leave the study, I may contact the Principal Investigator:

> Aaron Hogue, Ph.D.
> Partnership to End Addiction
> 711 Third Avenue, 5th Floor New York, NY 10017
> (212) 841-5278

If I have questions about my rights as a research participant or would like to report a negative event related to this study, I may contact Solutions IRB at (855) 226 – 4472 or by email at participants@solutionsirb.com.

I have read and understood this Consent Form and I agree to participate in this research study. I give Aaron Hogue, Ph.D. and his team permission to present this work in written and oral form, without further permission from me. I understand that I will be given a digital copy of this form.

Do you consent to participate in this study?
___Yes

___No

____ ____________________________________
Printed Name Signature

____
Date
