# Supplementary material for: Standard Versus Family-Based Screening, Brief Intervention, and Referral to Treatment for Adolescent Substance Use in Primary Care: Protocol for a Multisite Randomized Effectiveness Trial
Source: JMIR Res Protoc. 2024 May 31;13:e54486. doi: 10.2196/54486 (PMC11179044; doi:10.2196/54486)
Supplement: Multimedia Appendix 7 [file resprot_v13i1e54486_app7.docx]

**Study Committees/Teams**

**Principal Investigator**

Design and conduct of the trial

Preparation of protocol and revisions

Publication of study reports

**Steering Committee**

(see title page for members)

Approve final protocol

All lead investigators serve as steering committee members

Recruit clinics with the PI

Review study progress, and if necessary, approve changes to the protocol to facilitate study implementation.

**Data Manager Study Coordinator**

Maintenance of trial IT system and data entry

Data verification

**Local Champions & Site Research Coordinators**

In each participating clinic a liaison (program director/clinical supervisor) will be identified, to be responsible for coordinating training workshops and maintaining open communication with the study team regarding recruitment, data submission, technical assistance needs, and staff turnover and feedback.

**Data Safety and Monitoring Board**

An independent group of experts charged with reviewing study data for quality and integrity, adherence to the protocol, participant safety, study conduct and progress, and making determinations regarding study continuation, modifications, and suspensions/terminations will be appointed. The DSMB will work in conjunction with the PI and IRB.
