# Supplementary material for: Standard Versus Family-Based Screening, Brief Intervention, and Referral to Treatment for Adolescent Substance Use in Primary Care: Protocol for a Multisite Randomized Effectiveness Trial
Source: JMIR Res Protoc. 2024 May 31;13:e54486. doi: 10.2196/54486 (PMC11179044; doi:10.2196/54486)
Supplement: Multimedia Appendix 8 [file resprot_v13i1e54486_app8.docx]

**EXTERNAL:** This email originated from outside of the organization.

| 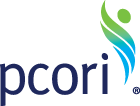 |
| --- |
|  |
| \| Request ID#: PIR-1639  Project Title: Adolescent-Only SBI versus Family-Based SBI in Primary Care for Adolescent Alcohol Use  **PRIVILEGED COMMUNICATION**  Dear Aaron Hogue:  Thank you for your interest in PCORI and for the submission of your application to the Cycle 1 2022 Brief Interventions for Adolescent Alcohol Use program.  Your application was discussed during the merit review panel. We are now conducting our preliminary programmatic and administrative review to assist us in evaluating your application. This email should not be construed as approval of the application budget or project plan; instead, it helps inform our decision on the final set of applications to recommend for funding.  During our review we have identified questions that require additional information related to the project plan and budget for your study.  The questions are listed in detail below. Please address the following issues using [PCORI Online](https://protect-us.mimecast.com/s/YEHdC1w4m2UxV2YtGUErL?domain=pcori.force.com) by **8/23/2022, 5:00 PM**.  **PROGRAMMATIC**  **Please respond to each of the below concerns raised by Merit Reviewers.**  **1.      Recruitment.**  **a.**Reviewers felt that the project’s recruitment projections and planned enrollment rate of roughly 5 new subjects per clinic, per week (who meet the eligibility criteria, and whose families provide consent) are overly optimistic, and lacked sufficient justification. Please respond to this concern and provide evidence of recruitment rates for similar trials in which you have been involved and from published literature, including recruitment by site, as appropriate.  **b.**Reviewers recommended that the research team have contingency plans in place to bring one or more sites on in case the planned enrollment rate is lower than expected.  **c.**Reviewers noted that the application could be more intentional in terms of recruiting racial and ethnic minority populations.  **2.      Primary outcome measure.** Reviewers noted that the selected primary outcome measure of ALEXSA/YRI has not been frequently used in research studies to date and that a better justification for use of these measures (as opposed to more typical measures) is needed. Additionally, it was noted that the use of this outcome measure appears to have been adapted from a larger measure and there was inadequate explanation for the reasoning behind this.    **3.      Fidelity monitoring**. Reviewers noted that the application lacked sufficient details and clarification regarding how fidelity monitoring will be assessed and carried out. Specifically, it was noted that the application stated that the count of number of interviews completed will be assessed. However, reviewers felt that this does not address the quality of interviews, nor did they see any benchmarks or how the research team will monitor fidelity besides the number of interviews which misses content.  **4.      Conceptual framework.** Reviewers noted that the conceptual framework was lacking in terms of being anchored in background literature to inform the selection of key variables and relationships between the interventions and outcomes, as well as in terms of cohesiveness.    **5.      Power analysis and analytic plan.** Reviewers were concerned that the power analysis described in the application does not match the analytic plan. Specifically, it was noted that the power analysis was conducted using a nested approach (i.e., participants nested within sites) with an ICC of .02 for sites; however, the analytic plan provides a justification for not using random effects for site.  **6.      Potential bias.** Reviewers noted that although the application addresses the limited chance of cross-contamination, there is still a chance it could happen given that both interventions will be implemented in each of the 3 sites.  **7.      Clarification.** The comparators are described as client facing but the application indicates that participants will have the choice of client-facing or provider facing services, and it is not clear how this will be incorporated to test hypotheses.  **8.      Patient engagement.** Reviewers noted that the application lacked inclusion of persons with lived experiences or peer recovery support specialists administering the tests to patients. It was felt that the inclusion of such individuals to administer testing would benefit the study as adolescents and caregivers would be more comfortable, open, and truthful.  **9.      Stakeholder engagement.** Reviewers noted that although the application mentions the involvement of the partner panel throughout the project, the engagement of each of the stakeholders appears to be fragmented, and meeting only twice a year may impact the active engagement process of the partner panel, such as preventing every partner on the panel from being involved in all phases of the study.  **ADMINISTRATIVE**  - No Administrative Requests -  **SUPPLEMENTAL DOCUMENTATION**  Please download and complete the Previous, Current, and Pending Support template available [here](https://protect-us.mimecast.com/s/PA5wC2kWn3hWMPwSB2Lcv?domain=pcori.org). This should be completed for all key personnel and returned as one Microsoft Word attachment in your response to PCORI.  **Please disregard the above supplemental documentation request if you have already submitted this information to the PCORI staff.  We recognize that a new budget may be required to respond appropriately to the programmatic concerns. If there is a significant impact on the budget, then you will need to provide a revised budget taking into account the suggested changes.  To access and respond to the PCORI Information Request, please follow the link provided below and log in to PCORI Online.  Access Your PCORI Information Request from the Following URL:  [https://pcori.force.com/engagement/s/detail/a3J5Y000003wuXo](https://protect-us.mimecast.com/s/w9M9CZ60l3FAJrNizIacr?domain=pcori.force.com)  **Guidelines for Submission**   1. Submit document(s) as attachments through PCORI Online only using the link provided. 2. Submit programmatic and administrative responses in separate documents. 3. Submit all response attachments at once using the **Submit Response**button in PCORI Online; do NOT reply to this email with your response and/or attachments. 4. BUDGET SUBMISSION: If an updated budget has been requested by PCORI, please use the budget template provided by PCORI. Please complete and upload as a Microsoft Excel file.   **Responding to the aforementioned requests does not guarantee that an application will be recommended for funding**. All awards recommended for funding will undergo a thorough business review before contract execution.  **PLEASE NOTE**   - **If applicable, under the PCORI funding contract, awardees are responsible for complying with all laws, including not violating any third party’s intellectual property rights.** - **We expect our awardees to obtain the proper licenses and permissions and adhere to the terms of the licenses for all third-party content used in connection with the awardee’s funded project.**   This email should be treated as confidential and not shared publicly or with others within the organization who are not involved with the preparation of response materials.  If you would like to discuss this letter, please email [pfa@pcori.org](mailto:pfa@pcori.org) with available times for a call, and we will coordinate a meeting.  Sincerely,  Contract Management  Program Support and Information Management  Patient-Centered Outcomes Research Institute \| \| --- \| |
|  |
| 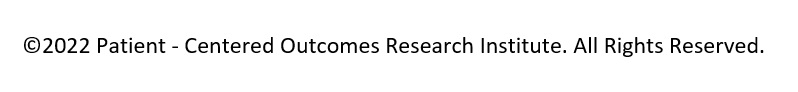 |
|  |
